# Maternal Depressive Symptoms across Early Childhood and Asthma in School Children: Findings from a Longitudinal Australian Population Based Study

**DOI:** 10.1371/journal.pone.0121459

**Published:** 2015-03-26

**Authors:** Rebecca Giallo, Salma Bahreinian, Stephanie Brown, Amanda Cooklin, Dawn Kingston, Anita Kozyrskyj

**Affiliations:** 1 Murdoch Childrens Research Institute, Parkville, Australia; 2 University of Alberta, Edmonton, Canada; 3 Judith Lumley Centre, La Trobe University, Victoria, Australia; Institute of Psychiatry, UNITED KINGDOM

## Abstract

There is a growing body of evidence attesting to links between early life exposure to stress and childhood asthma. However, available evidence is largely based on small, genetically high risk samples. The aim of this study was to explore the associations between the course of maternal depressive symptoms across early childhood and childhood asthma in a nationally representative longitudinal cohort study of Australian children. Participants were 4164 children and their biological mothers from the Longitudinal Study of Australian Children. Latent class analysis identified three trajectories of maternal depressive symptoms across four biennial waves from the first postnatal year to when children were 6–7 years: minimal symptoms (74.6%), sub-clinical symptoms (20.8%), and persistent and increasing high symptoms (4.6%). Logistic regression analyses revealed that childhood asthma at age 6–7 years was associated with persistent and increasing high depressive symptoms after accounting for known risk factors including smoking during pregnancy and maternal history of asthma (adjusted OR 2.36, 95% CI 1.61–3.45), p.001). Our findings from a nationally representative sample of Australian children provide empirical support for a relationship between maternal depressive symptoms across the early childhood period and childhood asthma. The burden of disease from childhood asthma may be reduced by strengthening efforts to promote maternal mental health in the early years of parenting.

## Introduction

Asthma is the most common chronic disease among children, and recognised by the World Health Organisation as a global public health concern [1,2]. Australia has one of the highest rates of childhood asthma in the world [3], with national survey data showing that 10–17% of Australian children (0–15 years) are affected [4]. Asthma has long-term effects on children’s overall health and quality of life, and is associated with considerable heath care resource use [4]. In Australia, health-care expenditure attributed to asthma was estimated to be AUS $606 million in 2004–05[4]. The rising prevalence of asthma over recent decades [5] means the costs are likely to increase unless ways are found to reduce the burden of disease.

Despite decades of medical research into the etiology of childhood asthma, understanding of the complex interplay of causal factors remains elusive. Although genetic and environment risk factors (e.g., family history of asthma, exposure to cigarette smoke, urban residence) have been implicated [6], none of these factors alone satisfactorily explains the high prevalence in high income countries. Recent research has begun to focus on understanding the psychobiological processes associated with early life exposure to adversity, which may threaten infant neurodevelopment and regulation of the hypothalamic-pituitary-adrenal (HPA) axis, increasing risks for poor health across the lifespan [7,8]. Evidence for the associations between childhood asthma and adversity suggests that some of the burden of disease from asthma could be avoided by addressing causes of health inequalities and modifiable risk factors [9]. One such factor is maternal mental health problems.

Depression and anxiety affect approximately 19% of women in the first year postpartum [10], and can persist into the early childhood period for many women [11,12]. Whilst earlier studies were instrumental in providing the first evidence for an association between postnatal depression and childhood asthma and potential biological pathways, they were based on relatively small, genetically high risk or inner-city cohorts in the US followed until preschool age [13] [14] [15] [16].

To date, three population-based studies have tested the association between perinatal distress and childhood asthma. Studies from the UK [17] and Netherlands [18] found that anxiety or depression during pregnancy, but not postnatal depression, was associated with childhood asthma and wheeze during the preschool years. Despite such findings, assessment of maternal mental health in the first postnatal year represents a critical window as children’s biological systems are rapidly maturing within the first two years of life, and may be compromised by exposure to more severe and enduring maternal mental health problems beyond the first postnatal year. This was observed in a population-based birth cohort of 13,907 Canadian urban and rural children, which found that continued exposure to maternal anxiety or depression from birth into early childhood, rather than exposure limited to first 12 months postpartum, was associated with a twofold increase in the risk of asthma at age 7 years [19]. Women with persistent mental health problems also had higher health care use postpartum. Whilst these findings suggest a dose response relationship between the chronicity of maternal distress and childhood asthma, the results were based on women with severe symptoms who sought services for mental health issues. The question remains whether the increased risk of asthma is primarily for children exposed to more severe symptoms, or whether the risk is also evident for children exposed to lower, subclinical levels of depressive symptoms, which are more common. This knowledge underlies accurate identification of children at risk for asthma.

To address gaps in current evidence, the present study sought to examine the relationship between both the *severity* and *chronicity* of maternal depressive symptoms across the early childhood period and prevalence of childhood asthma in a nationally representative longitudinal cohort study of Australian families. We aimed to examine the associations between trajectories of maternal depressive symptoms and childhood asthma at 6–7 years, whilst accounting for known risk factors for childhood asthma such as maternal history of asthma, smoking during pregnancy [6], socioeconomic disadvantage [9], and living in an urban metropolitan area [6].

## Materials and Methods

### Study design and sample

Data were drawn from waves 1–4 of the Growing up in Australia: Longitudinal Study of Australian Children (LSAC) Baby cohort. All aspects of the project design including recruitment and consent procedure was approved by the Australian Institute of Family Studies Human Research Ethics Committee. Informed written consent was obtained from parents and caregivers on behalf of the children enrolled in the study. The sampling design and field methods are detailed elsewhere [20], however, in short, a two-stage sampling design was used. First, 10% of all Australian postcodes (stratified by state of residence and urban versus rural) were selected. Next, a number of children proportional to the population size were randomly selected from each postcode using Australia’s universal health insurance (Medicare) database. Data on a broad range of child health, wellbeing and developmental domains, as well as parent and family functioning were obtained from primary and secondary caregivers via a combination of face-to-face interview and self-report questionnaires.

The B-cohort was recruited in 2004, consisting of 5107 children aged 3–12 months (wave 1; 64% response rate). Compared to the Australian population, children from non-English speaking families, single parents and those living in rental properties were slightly under-represented. Children and their families were followed up biennially when aged 2–3 (wave 2), 4–5 (wave 3) and 6–7 years (wave 4). The retention rate from wave 1–2, 2–3 and 3–4 was 90%, 86% and 84% respectively. Retention rates were lower for children whose parents had a lower educational attainment, were from Indigenous and non-English speaking backgrounds, and were living in rental properties [20].

The sample for the present study was biological mothers and their children who had more than 80% data available on the variables of interest from birth to age 6–7 years.

### Measures

*Childhood asthma* at age 6–7 years was defined as caregiver report of the presence of all three indicators: (a) having ever been diagnosed with asthma by a physician, (b) presence of wheeze and/or whistling, or an illness accompanied by wheezing in the previous 12 months, and (c) receipt of asthma medication in the previous 12 months. This definition accords with recommendations of the International Study of Asthma and Allergy in Childhood [21], and has been used in a number of previous studies [9].

*Maternal depressive symptoms* were assessed at all waves using the Kessler-6 (K6), a widely used screening tool for identifying mood and anxiety disorders based on DSM-IV criteria [22]. The six items assess symptoms such as feeling nervous, hopeless, restless, extreme are rated on a 5-point scale ranging from 0-None of the time to 5-All or most of the time. Items are summed, with higher scores indicating higher levels of psychological distress. Scores of 8–12 indicate elevated depressive symptoms, and scores of 13 or higher indicate probable clinical diagnosis of a mental health condition. Cronbach’s α for mothers in the current sample ranged from. 83 at wave 1 to. 86 at wave 4.

*Potential confounders* of the relationship between maternal depressive symptoms and childhood asthma were all assessed at wave 1 (age 3–12 months). These included child gender, maternal smoking during pregnancy, maternal use of asthma medication during pregnancy, instrumental delivery (caesarean, vacuum extraction and/or forceps), pre-term birth (<37 weeks), low birth weight (<2500 grams), not being breast fed, attending a child care centre within the first year of life, maternal age, number of children in the family, living in a metropolitan area, and low socio-economic position (SEP). SEP is a composite variable was derived by ranking each family’s relative SEP based on parental income, education and occupational prestige.15 Families with a standardised score at or below the 25th percentile were classified as ‘low’ whilst those above the 75th percentile were ‘high’ SEP, and the remainder ‘medium’ SEP.

### Data analysis

Analyses were conducted in two stages. First, latent class analysis (LCA) was conducted to identify trajectories of maternal depressive symptoms across four time points (3–12 months, 2–3, 4–5, and 6–7 years postpartum) using MPlus Version 7.11[23]. This involves identifying the smallest number of classes starting with a parsimonious 1-class model and fitting successive models with increasing numbers of classes. Model solutions were evaluated by inspecting whether the predicted number of classes approximated the distributions and patterns observed in these data, and by statistically by comparing Likelihood-ratio statistic (L^2^), Bayesian Information Criterion (BIC), and Akaike Information Criterion (AIC) across the successive models. Better fitting models have lower L^2^, BIC and AIC values. Entropy is an index for assessing the precision of assigning latent class membership, with higher probability values indicating greater precisions of classification. The Vuong-Lo-Mendall-Rubin Likelihood Ratio Test was also used to test for significant differences between the models. Class membership in the depressive symptom trajectories or all women in the sample were recorded and used in subsequent analyses.

Logistic regression was performed to investigate the associations between the maternal depressive symptom trajectories and childhood asthma whilst accounting for potential confounders such as maternal smoking, use of asthma and anti-depressant medication during pregnancy, child gender, instrumental delivery, prematurity, birth weight, breastfeeding, childcare attendance, socioeconomic status, single parenthood, maternal age, other siblings in the family, and urban or rural residency. To determine whether separate models should be tested for boys and girls, we examined the interaction term between child gender and maternal depressive symptom trajectories on childhood asthma. Results are presented as odd ratios with 95% confidence intervals. All analyses were weighted using wave 1 sample weights. Missing data were handled using multiple imputation. We imputed ten complete datasets under a multivariate normal model incorporating all analysis variables in SPSS V21.0 [24].

Finally, the regression analyses were re-run excluding 1183 children reported to have wheeze symptoms or illness with wheeze in the first year of life as a sensitivity analyses to increase our confidence that maternal depressive symptoms preceded the onset of childhood asthma rather than resulted from asthma symptomatology.

## Results

### Sample characteristics

At wave 1, 5107 children and their families were recruited into the LSAC study. Participant retention at wave 4 was approximately 86%. Of the 4242 children and their families still participating in the B-cohort at wave 4, 4164 met the inclusion criteria. Cases were excluded because the primary caregiver was not the biological mother (n = 64, 1.5%), or they had more than 20% missing data on the variables of interest (n = 14, 0.3%). There were significant differences between those included in the final sample and those excluded from the analyses on several demographic characteristics at p<.001. Mothers excluded from the analyses were more likely to be born outside Australia, from non-English speaking and Aboriginal Torres Strait Islander backgrounds, head a single parent family, and have a lower educational attainment compared to mothers in the final sample. After excluding the cases above missing data were minimal at <10%, except for smoking during pregnancy and depressive symptoms (K6 scores), which had 12.5% and 16.4% missing data, respectively.

There were 381 (9.1%) children in the sample who met the study criteria for asthma at age 6–7 years. Although the proportion of boys with asthma was greater than that of girls, the difference was not statistically significant (boys: n = 211, 9.8%; girls: n = 170, 8.4%; χ^2^(N = 4164) = 2.42, p = .066). Sample characteristics for children with and without asthma and the overall sample are presented in Table 1. Significance tests (chi-square tests for categorical variables and ANOVAs for continuous variables) revealed that compared to children without asthma, significantly more children with asthma came from a one parent family (p = .039); were from a low socioeconomic background (p = .017), had a younger mother (p = .010); had a mother who used antidepressants (p = .030) and asthma medication (p<.001) during pregnancy.

**Table 1:** Sample characteristics for children with and without asthma and the overall sample.

	Children with asthma(*n* = 381)	Children without asthma(*n* = 3784)	Total Sample(N = 4164)
*Maternal characteristics*			
Maternal age (M, SD)	30.66 (5.13)	31.37 (5.18)	31.31 (5.18)
Born in Australia	318 (83.5%)	3014 (79.7%)	3332 (80%)
English speaking	339 (89.0%)	3308 (87.4%)	3647 (87.6%)
Aboriginal and Torres Strait Islander	8 (2.1%)	86 (2.2%)	94 (2.2%)
Completed high school	120 (31.5%)	1111 (29.4%)	1231 (29.6%)
One parent family	38 (10.0%)	266 (7.0%)	304 (7.3%)
Low socioeconomic position	98 (25.7%)	773 (20.4%)	871 (20.9%)
Lived in metropolitan/urban area	236 (61.9%)	2367 (62.6%)	2603 (62.5%)
Smoked during pregnancy	59 (18.4%)	507 (15.3%)	566 (15.5%)
Antidepressant use during pregnancy	80 (2.1%)	15 (3.9%)	95 (2.3%)
Asthma medication during pregnancy	36 (9.4%)	112 (3.0%)	148 (3.6%)
Instrumental delivery	153 (40.2%)	1447 (38.3%)	1600 (38.4%)
*Child characteristics*			
Gender—Male	211 (55.4%)	1937 (51.2%)	2148 (51.6%)
Age in months at wave 1 (M, SD)	8.56 (2.54)	8.79 (2.57)	8.77 (2.57)
Pre-term (<37 weeks)	27 (7.1%)	238 (6.3%)	265 (6.4%)
Low birth weight (<2500 grams)	20 (5.3%)	194 (5.2%)	214 (5.2%)
Never breastfed	32 (8.4%)	266 (7.0%)	298 (7.2%)
Attended child care in first year of life	37 (9.7%)	441 (11.7%)	478 (11.5%)
First child	151 (39.6%)	1491 (39.4%)	1642 (39.4%)

### Trajectories of maternal depressive symptoms across the early childhood period

Mean K6 scores for waves 1–4 were 3.41 (SD = 3.33), 2.85 (SD = 3.12), 3.11 (SD = 3.24), and 3.15 (SD = 3.49), respectively. The proportion of mothers in the symptomatic range (K6 scores 8–12) indicating elevated distress at waves 1–4 were 7.6% (n = 317), 6.1% (n = 254), 6.1% (n = 254), and 8.0% (n = 333), respectively. The proportions of mothers in the clinical range (K6 scores 13+) at waves 1–4 were 1.8% (n = 77), 1.5% (n = 62), 1.8% (n = 73), and 2.4% (n = 102), respectively.

Latent class models specifying 1–4 trajectories were estimated (see Table 2). The 3-class model was accepted as the final model as its fit indexes (L^2^, BIC, AIC) were lower than the 1- and 2-class models. Furthermore, the Vuong-Lo-Mendell-Rubin Likelihood Ratio Test indicated a significant difference between the 2- and 3-class models, suggesting that the latter gives significant improvement in model fit over the 2-class model. The 4-class model was not selected as the difference between the 3- and 4-class models was not significant, and the purpose of LCA is to select the model with no more groups than is necessary to describe the distinct features of the data [25].

**Table 2:** Model fit indexes for latent classes of depressive symptoms from pregnancy to 4 years postpartum.

Model	L^2^	BIC	AIC	Entropy	Vuong-Lo-Mendell-Rubin Likelihood Ratio Test *p*	
1-class	-38972.25	78011.18	77960.50	-	-	-
2-class	-36924.48	73957.29	73874.95	.90	1 vs 2 classes	<.001
3-class	-36352.70	72855.42	72741.40	.86	2 vs 3 classes	<.001
4-class	-36038.55	72268.78	72123.09	.88	3 vs 4 classes	. 064

Note: L^2^ = Likelihood-ratio statistic, BIC = Bayesian Information Criterion, AIC = Akaike Information Criterion

**Fig 1:**
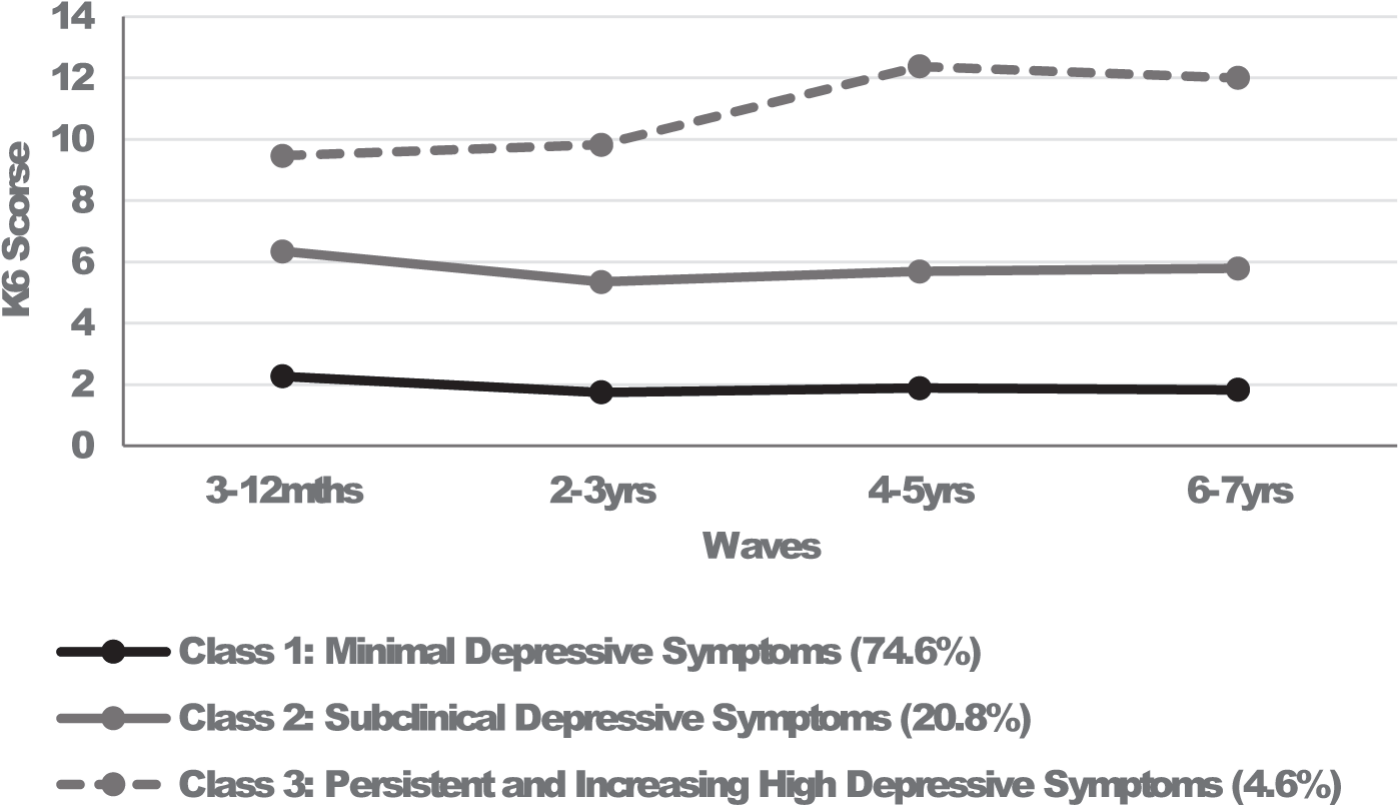
Estimated means on the K6 for the trajectories of maternal depressive symptoms across the early childhood period.

The entropy value for the 3-class model was high (.86), suggesting acceptable precision in assigning individual cases to their appropriate class. The average posterior probabilities were acceptable for Class 1 (.96), Class 2 (.86), and Class 3 (.94). Fig. 1 illustrates the three trajectory classes of maternal depressive symptoms. The first and largest trajectory consisted of women who reported ‘minimal depressive symptoms’ from 3–12 months to 6–7 years postpartum (n = 3105, 74.6%, K6 scores ranging 1.8–2.2). The second trajectory consisted of women who reported ‘subclinical depressive symptoms’ over time (n = 868, 20.8%, K6 scores ranging 5.3–6.3). The smallest trajectory consisted of women who reported ‘increasing and persistently high depressive symptoms’ above the symptomatic cut point of 8 at all waves (n = 191, 4.6%, K6 scores ranging 9.5–12.4).

The sample characteristics for mothers assigned to the minimal, subclinical and persistent and increasing high symptoms are presented in Table 3. Mothers assigned to the subclinical and persistent and increasing high symptoms classes were more likely to have smoked (p<.001) and used antidepressant medication (p<.001) during pregnancy, be primiparous (p<.001), not completed high school (p<.001), be born in Australia (p<.001), English speaking (p<.001), from an Aboriginal or Torres Strait Islander background (p<.001); from a lower SEP (p<.001), be heading a single parent household (p<.001) and be younger in age (p<.001).

**Table 3:** Sample characteristics for mothers with minimal, subclinical and persistent and increasing high depressive symptoms.

	Minimal depressive symptoms(*n* = 3097)	Subclinical depressive symptoms(*n* = 868)	Persistent and increasing high depressive symptoms(N = 191)
*Maternal characteristics*			
Maternal age (M, SD)	31.49 (5.12)	30.95 (5.13)	30.03 (6.10)
Born in Australia	2529 (81.4%)	655 (75.5%)	148 (77.5%)
English speaking	2771 (89.2%)	728 (83.9%)	148 (77.5%)
Aboriginal and Torres Strait Islander	67 (2.1%)	14 (1.6%)	13 (6.8%)
Completed high school	877 (28.3%)	269 (31.0%)	85 (44.5%)
One parent family	179 (5.8%)	80 (9.2%)	45 (23.6%)
Low socioeconomic position	599 (19.3%)	192 (22.1%)	80 (41.9%)
Lived in metropolitan/urban area	1912 (61.6%)	569 (65.6%)	122 (63.9%)
Smoked during pregnancy	364 (13.2%)	156 (20.7%)	46 (32.6%)
Antidepressant use during pregnancy	38 (1.2%)	39 (4.5%)	18 (9.4%)
Asthma medication during pregnancy	109 (3.5%)	28 (3.2%)	11 (5.8%)
Instrumental delivery	1190 (38.3%)	348 (40.1%)	62 (32.5%)
*Child characteristics*			
Gender—Male	1598 (51.5%)	444 (51.2%)	106 (55.5%)
Age in months at wave 1 (M, SD)	8.78 (2.52)	8.74 (2.68)	8.64 (2.70)
Pre-term (<37 weeks)	187 (6.0%)	60 (6.9%)	18 (9.5%)
Low birth weight (<2500 grams)	152 (4.9%)	48 (5.6%)	14 (7.4%)
Never breastfed	222 (7.1%)	55 (6.3%)	21 (11.0%)
Attended child care in first year of life	358 (11.5%)	101 (11.6%)	19 (9.9%)
First child	1213 (39.1%)	354 (40.8%)	75 (39.3%)

### Maternal depressive trajectories and childhood asthma at age 6–7 years

To determine whether separate models for boys and girls should be estimated, the interaction term between maternal depressive trajectories and child gender on asthma outcome was assessed. It was found to be non-significant, so we estimated the models for the overall sample.

Without adjusting for potential confounders, we found that the persistent and increasing high depressive symptoms trajectory was associated with a 2.81 increased odds of childhood asthma at aged 6–7 years. In the final multivariable model (Table 4) persistent and increasing high depressive symptoms remained significantly associated with childhood asthma (adjusted OR = 2.36, p<.001), after adjusting for the factors significantly associated with asthma in the bivariate analyses. In addition to persistent and increasing high depressive symptoms, the strongest predictors of asthma were maternal asthma and antidepressant medication during pregnancy.

**Table 4:** Bivariate and multivariable results for factors associated with asthma at 6–7 years.

	BivariateOR (95% CI), *p*	MultivariableOR (95% CI), *p*
Maternal depressive trajectories		
Minimal symptoms	Reference	Reference
Subclinical symptoms	1.23 (0.95–1.58),. 121	1.16 (0.89–1.51),. 266
Persistent and increasing high symptoms	2.81 (1.96–4.03), <.001	2.36 (1.61–3.45), <.001
Smoking during pregnancy	1.35 (1.02–1.79),. 037	1.09 (0.80–1.47),. 600
Asthma medication during pregnancy	3.45 (2.33–5.12), <.001	3.28 (2.12–5.06), <.001
Anti-depressant medication during pregnancy	2.38 (1.41–4.00),. 001	1.77 (1.02–3.07),. 042
Child gender—male	1.19 (0.96–1.47),. 108	-
Instrumental delivery	1.08 (0.87–1.35),. 463	-
Pre-term birth (<37 weeks)	1.15 (0.77–1.72),. 487	-
Low birthweight	0.95 (0.59–1.52),. 818	-
Not breastfed	1.33 (0.93–1.89),. 115	-
Attends child care centre	0.81 (0.56–1.16),. 242	-
Maternal age	0.98 (0.96–0.99),. 013	0.98 (.96–1.00),. 112
One parent family	1.54 (1.10–2.15),. 011	1.14 (0.78–1.67),. 495
Number of children in the family	0.98 (0.88–1.08),. 671	-
Low socioeconomic position	1.39 (1.10–1.75),. 006	1.15 (0.88–1.50),. 307
Living in metropolitan area	1.01 (0.80–1.26),. 959	-

### Sensitivity analyses

To increase our confidence that maternal depressive symptoms preceded the onset of childhood asthma rather than resulted from asthma symptomatology, the regression analyses were re-run excluding 1183 children reported to have wheeze symptoms or illness with wheeze in the first year of life. Of the 2909 children in the selected sample, 210 (7.2%) met the study criteria for asthma at 6–7 years. The pattern of results were similar to the main analyses, whereby children exposed to a trajectory of persistent and increasing high depressive symptoms across the early childhood period had an almost threefold increased risk of childhood asthma compared to children exposed to minimal depressive symptoms (see Table 5).

**Table 5:** Bivariate and multivariable results for factors associated with asthma at 6–7 years for children who did not have symptoms of wheeze in the first year of life.

	BivariateOR (95% CI), *p*	MultivariableOR (95% CI), *p*
Maternal depressive trajectories		
Minimal symptoms	Reference	Reference
Subclinical symptoms	1.31 (0.94, 1.85),. 111	1.33 (0.94–1.87),. 108
Persistent and increasing high symptoms	2.81 (1.68, 4.69), <.001	2.70 (1.59–4.59), <.001
Smoking during pregnancy	1.18 (0.79–1.77),. 414	1.02 (0.66–1.56),. 940
Asthma medication during pregnancy	3.342 (1.95–6.00), <.001	3.46 (1.96–6.11), <.001
Anti-depressant medication during pregnancy	1.46 (0.62–3.46),. 388	1.03 (0.42–2.54),. 944
Child gender—male	1.26 (0.95–1.67),. 107	-
Instrumental delivery	1.09 (0.82, 1.46),. 536	-
Pre-term birth (<37 weeks)	0.72 (0.37–1.41),. 358	-
Low birthweight	0.71 (0.34–1.50),. 374	-
Not breast fed	0.93 (0.53–1.63),. 796	-
Attends child care centre	0.67 (.39–1.16),. 153	-
Maternal age	0.97 (0.95–1.00),. 059	0.98 (0.95–1.01),. 110
One parent family	1.20 (0.71–2.03),. 499	0.89 (0.50–1.60),. 697
Number of children in the family	0.89 (0.77–1.04),. 134	-
Low socioeconomic position	1.28 (0.93–1.76),. 132	1.13 (0.79–1.62),. 502
Living in metropolitan area	0.94 (0.70–1.26),. 682	-

## Discussion

This is the first study to examine the long-term associations between the severity and course of maternal depressive symptoms across early childhood period and childhood asthma in a large contemporary population-based sample of Australian children and their families. We found that exposure to persistent and increasing maternal depressive symptoms were associated with approximately a threefold increased odds of asthma at 6–7 years (crude OR = 2.81, 95% CI, 1.68–4.69). The magnitude of the association remained similar, and highly significant even with adjustment for known factors associated with childhood asthma such as maternal history of asthma (as measured by asthma medication use during pregnancy), maternal smoking during pregnancy and other socio-contextual factors (adjusted OR = 2.70, 95% CI, 1.59–4.69). Our findings improve upon previous studies solely focused on the postnatal period in high risk genetic samples, highlighting that maternal depressive symptoms in the first 12 months postpartum and *beyond* is a key risk factor for childhood asthma.

We did not find an association between sub-clinical levels of maternal depressive symptoms and childhood asthma, suggesting a dose response where children of severely depressed mothers whose mental health worsens over time are at greater risk of developing childhood asthma. One possible mechanism for the observed relationships between maternal depression and childhood asthma in our study is via neuroregulatory dysfunction of the HPA axis. Several studies have shown that maternal postnatal depression and anxiety strongly correlate with infant cortisol levels [26,27]. HPA dysregulation and the production of proinflammatory cytokines may increase children’s susceptibility and sensitivity to persistent maternal distress and other co-occuring sources of chronic stress across the early childhood period [27,28]. This may manifest as high allostatic load or a blunted cortisol response, heightening children’s vulnerability for the development of asthma [7,19,29].

### Study Strengths, Limitations and Future Directions

This study improves upon previous cross-sectional research based on small samples of children with high genetic risk for asthma by replicating findings with a nationally representative contemporary cohort of Australian families. We used sophisticated longitudinal modeling to describe both the *severity* and the *chronicity* of mothers’ depressive symptoms across the formative years of their children’s lives. We also controlled for potential bias associated with known factors for asthma (i.e., maternal history of asthma, smoking during pregnancy, socio-economic status), and found that the associations between maternal depressive symptoms and asthma were not attenuated. Finally, we addressed issues related to temporality by conducting a sensitivity analysis for a subset of the sample who did not have wheeze symptoms in their first year of life. The similar pattern of results strengthened our confidence that maternal depressive symptoms preceded the onset of childhood asthma rather than resulted from their child’s asthma symptomatology.

Despite these study strengths, there are some limitations to note. Considering the composition of study participants, generalisability of the findings to families from non-English speaking, Aboriginal or Torres Strait Islander, and lower socioeconomic background may be limited. The prevalence of asthma among Indigenous children [30] and those from low socioeconomic backgrounds [9] is higher than in the general Australian population, and it is likely that this study under- rather than overestimates—the associations between maternal depressive symptoms and asthma. Further research to examine these associations among specific population groups is needed. Although a broad range of potential confounding variables was accounted for, our analyses were limited by the measures collected in LSAC. Other variables worthy of future consideration, but not available in the LSAC dataset include maternal mental health during pregnancy, maternal use of antibiotics and infections during pregnancy. Similarly, directions for future research include identifying other early life factors such as family life stress, social support, and parent caregiving practices, which may mediate or moderate the effects of maternal distress on asthma outcomes for children. This work is needed to understand how optimal early caregiving environments may buffer or ameliorate the potential contribution of poor maternal mental health to children’s asthma.

### Policy and Practice Implications

Childhood asthma continues to be a global public health concern despite decades of medical research into its causes and treatment. Our study addresses the call from the World Health Organization to investigate the social determinants and psychobiological pathways underpinning the development and maintenance of non-communicable chronic health diseases over the life course [31]. We contribute to evidence that asthma is influenced by early and cumulative exposure to early life stress and social adversity in particular maternal depressive symptoms. This evidence suggests that a range of very different approaches are needed to supplement current medical approaches to asthma prevention, early intervention and treatment that have had limited effectiveness.

Current approaches to the treatment of childhood asthma primarily focus on (a) the pharmacological management of symptoms (i.e., inhaled corticosteroids), (b) non-pharmacological strategies to reduce modifiable risk factors (i.e., minimizing exposure to cigarette smoking and known allergens), and (c) strengthening the self-management skills of adolescents to manage their condition or those of caregivers’ to manage the symptoms of younger children [32]. Our findings suggest that an integrated health care approach to the management of childhood asthma is needed, where the medical, mental and social health care needs of children and families are addressed in primary health care and pediatric settings. Psychosocial interventions to address family adversity and maternal mental health may improve treatment adherence, reduce the burden of chronic disease, and save on health costs attributable to asthma related care.

Our findings also have particular implications for policy and practice efforts focused on asthma prevention. Emerging evidence suggests that early intervention efforts aimed at reducing psychobiological risk associated with early life adversity are effective in reducing neonatal morbidity [33]. Therefore, it is likely that mental health surveillance, early identification, and enhanced maternity and primary care support for women experiencing mental health difficulties and social adversity will be effective in reducing the psychobiological risks for childhood asthma and other non-communicable chronic health conditions. Ensuring that women and children have timely access to integrated medical, mental and social health care in the critical early years will maximize their potential for good health, wellbeing and quality of life across the life span.
